# Development and internal validation of simplified predictive scoring (ICU-SEPSA score) for mortality in patients with multidrug resistant infection

**DOI:** 10.3389/fphar.2022.938028

**Published:** 2022-08-26

**Authors:** Taranee Sirichayanugul, Chansinee Srisawat, Chawin Thummakomut, Abhisit Prawang, Nina S Huynh, Surasak Saokaew, Pochamana Phisalprapa, Sukrit Kanchanasurakit

**Affiliations:** ^1^ Division of Drug Information, Department of Pharmacy, Phrae Hospital, Phrae, Thailand; ^2^ Division of Clinical Pharmacy, Department of Pharmaceutical Care, School of Pharmaceutical Sciences, University of Phayao, Phayao, Thailand; ^3^ Department of Pharmacy Practice, Faculty of Pharmaceutical Sciences, Chulalongkorn University, Bangkok, Thailand; ^4^ College of Pharmacy, Rangsit University, Phathum Thani, Thailand; ^5^ Department of Pharmacy Practice, University of Illinois at Chicago, Chicago, IL, United States; ^6^ Division of Social and Administration Pharmacy, Department of Pharmaceutical Care, School of Pharmaceutical Sciences, University of Phayao, Phayao, Thailand; ^7^ Center of Health Outcomes Research and Therapeutic Safety (Cohorts), School of Pharmaceutical Sciences, University of Phayao, Phayao, Thailand; ^8^ Unit of Excellence on Clinical Outcomes Research and IntegratioN (UNICORN), School of Pharmaceutical Sciences, University of Phayao, Phayao, Thailand; ^9^ Novel Bacteria and Drug Discovery Research Group, Microbiome and Bioresource Research Strength, Jeffrey Cheah School of Medicine and Health Sciences, Monash University Malaysia, Bander Sunway, Malaysia; ^10^ Biofunctional Molecule Exploratory Research Group, Biomedicine Research Advancement Centre, School of Pharmacy, Monash University Malaysia, Bander Sunway, Malaysia; ^11^ Division of Ambulatory Medicine, Department of Medicine, Faculty of Medicine Siriraj Hospital, Mahidol University, Bangkok, Thailand; ^12^ Division of Pharmaceutical Care, Department of Pharmacy, Phrae Hospital, Phrae, Thailand

**Keywords:** mortality, multidrug-resistant infection, screening tool, antibiotic resistant, predictive scoring

## Abstract

**Background:** Mortality from multidrug-resistant (MDR) pathogens is an urgent healthcare crisis worldwide. At present we do not have any simplified screening tools to predict the risk of mortality associated with MDR infections. The aim of this study was to develop a screening tool to predict mortality in patients with multidrug-resistant organisms.

**Methods:** A retrospective cohort study to evaluate mortality risks in patients with MDR infections was conducted at Phrae Hospital. Univariable and multivariable analyses were used to classify possible risk factors. The model performance was internally validated utilizing the mean of three measures of discrimination corrected by the optimism using a 1000-bootstrap procedure. The coefficients were transformed into item scores by dividing each coefficient with the lowest coefficient and then rounding to the most adjacent number. The area under the receiver operating characteristic curve (AuROC) was used to determine the performance of the model.

**Results:** Between 1 October 2018 and 30 September 2020, a total of 504 patients with MDR infections were enrolled. The ICU-SEPSA score composed of eight clinical risk factors: 1) immunocompromised host, 2) chronic obstructive pulmonary disease, 3) urinary tract infection, 4) sepsis, 5) placement of endotracheal tube, 6) pneumonia, 7) septic shock, and 8) use of antibiotics within the past 3 months. The model showed good calibration (Hosmer-Lemeshow χ2 = 19.27; *p-*value = 0.50) and good discrimination after optimism correction (AuROC 84.6%, 95% confidence interval [Cl]: 81.0%–88.0%). The positive likelihood ratio of low risk (score ≤ 5) and high risk (score ≥ 8) were 2.07 (95% CI: 1.74–2.46) and 12.35 (95% CI: 4.90–31.13), respectively.

**Conclusion:** A simplified predictive scoring tool wad developed to predict mortality in patients with MDR infections. Due to a single-study design of this study, external validation of the results before applying in other clinical practice settings is warranted.

## Introduction

The burden of infections caused by multidrug-resistant organisms (MDROs) is increasing worldwide, while the available antibiotics become less effective over time. (Morrison and Zembower, 2020) Fifteen new antibiotics have been developed and launched in the market in a recent year, but only two of them can be effectively used to treat multidrug resistant gram-negative bacterial infections. (Carlet et al., 2012) Consequently, bacterial antimicrobial resistance is a critical and urgent problem that needs to be addressed globally. In 2019, it was estimated that the highest rate of antimicrobial-resistant burden was found in sub-Saharan Africa. Approximately 27.3 deaths per 100,000 population had been reported in the region. Other affected areas with high incidences included South Asia, Europe, Latin America, and Oceania. Most common organisms leading to deaths associated with resistance were 1) *Pseudomonas aeruginosa*, 2) *Acinetobacter baumannii*, 3) *Escherichia coli*, 4) *Staphylococcus aureus*, 5) *Streptococcus pneumoniae*, and 6) *Klebsiella pneumoniae*. Predominantly, respiratory infections, bloodstream infections, and intra-abdominal infections were among the most common infectious diseases associated with antimicrobial-resistance. (Murray et al., 2022) According to collective evidence from 1,023 hospitals in Thailand in 2010, almost one-third of hospitalized patients diagnosed with infectious diseases had multidrug-resistant (MDR) infections. The previous report revealed that *A. baumannii* (57.6%) and *P. aeruginosa* (20%–30%) were the leading organisms of MDR infections, respectively. (Anudit et al., 2016) Additionally, infections with gram-negative bacteria (i.e., *Enterobacteriaceae*) are crucial challenges in any health-care settings. It is widely recognized that they have become difficult to treat and often require a complex treatment plan. Despite optimal and aggressive therapy, they frequently lead to increased morbidity and mortality, resulted in heightening anxiety worldwide. (Kanchanasurakit et al., 2020).

MDR infections are associated with many problems including prolonged length of hospital stays, increased cost of treatment and mortality rates. (Cosgrove, 2006; Sunenshine et al., 2007; Tanwar et al., 2014; Lim et al., 2016; Founou et al., 2017) The previous studies have shown a relationship between higher rates of MDROs and mortality. (Lim et al., 2016; Mave et al., 2017; Teerawattanapong et al., 2018) The actual causes for this association are not well defined. Comorbidities and altered immune response of patients may also contribute to the increased risk of mortality. Nevertheless, other factors including the patient’s clinical status and source or severity of infection may have played a pivotal role. (Gandra et al., 2019) At present, we do not have any tools to predict the risk of mortality in these patients.

To the extent of our knowledge, we believe that a tool used for prediction of mortality in patients with MDR infections has not been established. The previous study has identified a risk scoring system to predict risk of acquiring MDR infection, however mortality outcome was not addressed. (Tseng et al., 2017) While there were tools to predict mortality in patients diagnosed with bacteremia, those tools did not specifically focus on patients with MDROs. (Shapiro et al., 2003, 2007; Taniguchi et al., 2017) The objective of this study was to develop a risk scoring model to predict mortality for patients with MDR infections.

## Materials and methods

### Setting

This study was carried out at Phrae Hospital, a 500-bed medical center, located in the northern part of Thailand.This work followed the Transparent Reporting of a multivariable prediction model for Individual Prognosis Or Diagnosis (TRIPOD) Statement (Supplementary Table S1). (Moons et al., 2015).

### Study design and study population

We conducted a single-center, retrospective cohort study of hospitalized adults (aged ≥ 18 years) with at least one positive culture of MDROs during the admission at Phrae Hospital between 1 October 2018, and 30 September 2020. A total of 504 patients were enrolled in the study. For individuals with multiple positive culture results within the same admission, we only used the data from their first result for analysis. Patients with negative culture results as well as those with positive culture results with mono resistance or susceptible to antibiotics were excluded. In addition, patients with missing data in the medical record were excluded.

Multidrug resistant bacteria was defined as acquired non-susceptibility to at least one agent in three or more antimicrobial categories. (Falagas and Karageorgopoulos, 2008; Magiorakos et al., 2012) Antibiotics are classified into one of the following classes: aminoglycosides, penicillins, penicillins plus beta-lactamase inhibitors, cephalosporins, fluoroquinolones, tetracyclines, macrolides, carbapenems, folate pathway inhibitors, glycopeptides, polymyxins, and lincosamides. (Magiorakos et al., 2012) In this study, the infectious disease physician diagnosed and registered patients with MDR infections depending on susceptibility tests and clinical manifestations.

### Data collection and definition

Demographic characteristics including age, sex, status of patients, receipt of intensive care unit (ICU) admission, duration of hospitalization, comorbidities, source of infection, risk factors for MDR, disease severity, and use of medical devices were collected. Comorbidities were identified according to the International Classification of Diseases, 10th Revision, Clinical Modification (ICD-10) code version 2016. These were: essential hypertension (I10), diabetes mellitus type 2 (E11.0-E11.9), dyslipidemia (E78.5), heart failure (I50.0-I50.9), chronic kidney disease (N18.3-N18.9), atrial fibrillation (I48.0-I48.9), chronic obstructive pulmonary disease (J44.9), gout (M10.9), liver cirrhosis (K74.6), coronary artery disease (I20.0, I21.3-I21.4, I25.2), epilepsy (G40.9), Alzheimer’s disease (G30.9), Parkinson’s disease (G20), cerebrovascular disease including stroke (I63.0-I63.9), depression (F32.9), anxiety (F41.1-41.2, 41.9), and schizophrenia (F20.9).

Immunocompromised hosts were defined as patients with active cancer, current use of chemotherapy or immunosuppressant drugs, Acquired Immune Deficiency Syndrome (AIDS), chronic alcohol use, dialysis including hemodialysis and continuous ambulatory peritoneal dialysis, and uncontrolled diabetes mellitus with HbA1C >7%. (Delves and Roitt, 2000) Sepsis and septic shock were identified according to the Sepsis-3 criteria. (Singer et al., 2016) Sepsis was defined as life-threatening organ dysfunction caused by a dysregulated host response to infection. For clinical operationalization, organ dysfunction can increase the Sequential Organ Failure Assessment (SOFA) score of 2 points or more. Septic shock can be clinically identified if patients were in need of vasopressor to maintain a mean arterial pressure of 65 mmHg or greater and serum lactate level greater than 2 mmol/L (>18 mg/dl) in the absence of hypovolemia (Kanchanasurakit et al., 2021). In this study, mortality was defined as an in-hospital mortality secondary to infections. The cause of death was confirmed by physicians specialized in infectious disease.

### Statistical analysis

Baseline characteristics are reported using frequency (percentage), mean (SD), or median (interquartile range) as appropriate. To analyze mortality associated with MDROs-infected patients, we conducted univariable analysis using chi-square or Fisher’s exact test. Odds ratios (ORs) and 95% confidence intervals (95%CI) were evaluated for each factor. Variables with *p-*value ≤ 0.1 were then selected into the multivariable model using logistic regression. Any risk factors with *p*-value < 0.05 were integrated in the process of developing the risk prediction model for mortality. Additionally, Akaike information criterion (AIC) and Bayesian information criteria (BIC) were used to help identify variables that would be incorporated in the predictive model. The model with the lowest AIC and BIC values was chosen. (Chakrabarti and Ghosh, 2011) Model performance in the original sample was evaluated using the C-statistic, Hosmer-Lemeshow statistic, calibration slope, and Brier score (Huang et al., 2020).

### Sample size

We calculated sample size by applying 20 outcome events per predictor variable (EPV). (Austin and Steyerberg, 2017) Based on the previous study that used five binary variables (Zhou et al., 2019), at least 100 patients with an outcome are required.

### Model internal validation

Internal validation was performed using a bootstrap procedure with 1,000 bootstrapped samples. We calculated the bootstrap performance (i.e., calibration slope, calibration-in-the-large [CITL] and C-statistic) of the final model to derive an optimism adjusting factor to correct the model for overfitting. To develop a scoring system, each variable was assessed and turned into a point using their weighted coefficients. These were then rounded to the most adjacent number to be used as a score.

### Score development

To derive the point-based system, optimism adjusted coefficient and the intercept from the final multivariable model were used. We weighted and transformed each coefficient by dividing it by the lowest coefficient in the model and rounding the results to the most adjacent number. (Harrell, 1996; Moons et al., 2002) To examine the discriminative power of the predictive score we developed, a receiver operating characteristic (ROC) curve was plotted and a Hosmer-Lemeshow goodness-of-fit test was performed. (Steyerberg et al., 2010) The cut-off score was chosen to classify patients into three groups based on the risk of mortality: low (≤ 49.54%), moderate (56.98%–81.42%), and high (≥ 85.53%), respectively. The sensitivity, specificity, positive predictive value (PPV), negative predictive value (NPV), accuracy, positive likelihood ratio (LR+), and negative likelihood ratio (LR-) were assessed. (Leisenring and Pepe, 1998; Janssens et al., 2005) The multi-collinearity was obtained using the variance inflation factor (VIF).

### Score validation

The score was internally validated using bootstrapping. A total of 1,000 bootstrap samples were drawn and the final model containing all included variables was fitted in each bootstrap sample. This process was followed by re-simplification of the score and point assignment using the logistic regression model in each bootstrap sample. Performance of the re-fitted, simplified score was appraised in each bootstrap sample and in the original sample using the performance measures described above. Mean bootstrap and bootstrap test performances were assessed. Subsequently, optimism and optimism-corrected performance estimates were calculated.

## Results

### Characteristics of patients

**FIGURE 1:**
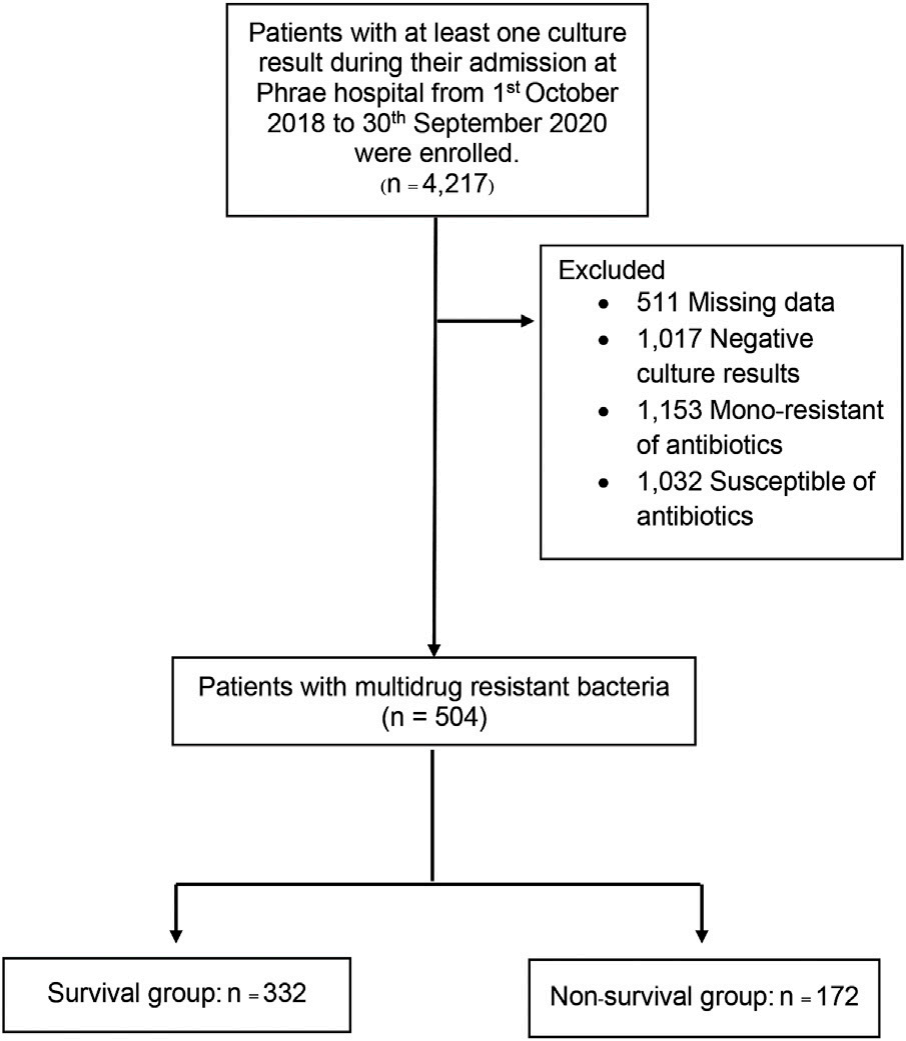
Enrollment of study patients.

From, 1 October 2018, through 30 September 2020, a total of 4,217 patients with culture results were identified during the study period. After excluding 3,713 patients with negative culture results, positive culture results with mono-resistance to antibiotics, missing data, or susceptible of antibiotics, 504 patients were included in the final analysis. There were 172 deaths (35%) during the follow-up (Figure 1). The mean age of the patients were 65 years, and the majority of patients (58%) were male. The mean duration of hospitalization was 18.39 days. Compared between the survival and non-survivor groups, there was no difference in bedridden status and comorbid conditions, except for heart failure, atrial fibrillation, and chronic obstructive pulmonary disease. Patients that died had recent admission at other facilities and later transferred to Phrae Hospital, admitted in the ICU, known immunocompromised status, and had recent antibiotic use with the past 3 months. Both groups had similar reports of wound infection. The survival group had less severe infections as well as fewer uses of medical devices. Baseline characteristics of both groups are shown in Table 1.

**TABLE 1:** Baseline characteristics of patients with MDROs.

Characteristics	Survival (*n* = 332)	Death (*n* = 172)	Total (*n* = 504)	*p*-value
Age (years), mean (±SD)	64.6 (15.1)	66.5 (14.5)	65.2 (15.0)	0.56
Male sex, No (%)	187 (56.3)	106 (61.6)	293 (58.13)	0.25
Duration of hospitalization, median (interquartile range), days	9 (5–20.5)	15 (7–28.5)	11 (5–23)	<0.001
Status of patients, No (%)				
ICU admission	75 (22.59)	57 (33.14)	132 (26.19)	0.011
Referral from other hospital	136 (40.96)	96 (55.81)	232 (46.03)	<0.001
Bedridden patients	42 (12.65)	28 (16.28)	70 (13.89)	0.26
Immunocompromised host	115 (34.64)	83 (48.26)	198 (39.29)	<0.001
Inter-current conditions, No (%)				
Hypertension	161 (48.49)	89 (51.74)	250 (49.60)	0.49
Type 2 Diabetes mellitus	89 (26.81)	47 (27.33)	136 (26.98)	0.90
Dyslipidemia	68 (20.48)	29 (16.86)	97 (19.25)	0.33
Heart failure	7 (2.11)	12 (6.98)	19 (3.77)	<0.001
Chronic kidney disease	88 (26.51)	50 (29.07)	138 (27.38)	0.54
Atrial fibrillation	12 (3.61)	19 (11.05)	31 (6.15)	0.001
Chronic obstructive pulmonary disease	20 (6.02)	26 (15.12)	46 (9.13)	0.001
Gout	33 (9.94)	10 (5.81)	43 (8.53)	0.13
Liver cirrhosis	9 (2.71)	7 (4.07)	16 (3.17)	0.41
Coronary artery disease	20 (6.02)	9 (5.23)	29 (5.75)	0.72
Neurological disease	7 (2.11)	7 (4.07)	14 (2.78)	0.20
Cerebrovascular disease	30 (9.04)	14 (8.14)	44 (8.73)	0.74
Mental disorder	6 (5.3)	2 (2.7)	8 (1.59)	0.58
Source of infection, No (%)				
Bacteremia	241 (72.59)	79 (45.93)	320 (63.49)	<0.001
Urinary tract	48 (14.46)	46 (26.74)	94 (18.65)	0.001
Pneumonia	14 (4.22)	41 (23.84)	55 (10.91)	<0.001
Intra-abdominal	0 (0)	5 (2.91)	5 (0.99)	0.002
Wound	29 (8.73)	13 (7.56)	42 (8.33)	0.65
Treatment history, No (%)				
Hospitalization within the past 3 months	142 (42.77)	86 (50.00)	228 (45.24)	0.12
Surgery within the past 3 months	26 (7.83)	9 (5.23)	35 (6.94)	0.28
Use of antibiotics within the past 3 months	120 (36.14)	86 (50.00)	206 (40.87)	0.003
Severity of infection, No (%)				
Sepsis	130 (39.16)	113 (65.70)	243 (48.21)	<0.001
Septic shock	42 (12.65)	68 (39.53)	110 (21.83)	<0.001
Medical devices, No (%)				
Foley catheter	148 (44.58)	133 (77.33)	281 (55.75)	<0.001
Endotracheal tube	76 (22.89)	116 (67.44)	192 (38.10)	<0.001

Abbreviations: ICU, intensive care unit; SD, standard deviation.

### Predictors for mortality in patients with multidrug-resistant organisms

Univariate analysis for mortality showed a significant association with hospitalization more than 14 days (OR 1.88, 95% CI: 1.27–2.78, *p-*value = 0.001), admission at ICU (OR 1.70, 95% CI: 1.10–2.61 *p-*value = 0.014), immunocompromised host (OR 1.76, 95% CI: 1.19–2.60, *p-*value = 0.004), heart failure (OR 3.48, 95% CI: 1.23–10.62, *p-*value = 0.012), atrial fibrillation (OR 3.31, 95% CI: 1.48–7.67, *p-*value = 0.002), chronic obstructive pulmonary disease (OR 2.78, 95% CI: 1.44–5.42, *p-*value = 0.002), bacteremia (OR 0.32, 95% CI: 0.21–0.48, *p*-value< 0.001), urinary tract infection (OR 2.16, 95% CI: 1.33–3.49, *p-*value = 0.001), pneumonia (OR 7.11, 95% CI: 3.63–14.56, *p-*value< 0.001), use of antibiotics within the last 3 months (OR 1.77, 95% CI: 1.19–2.61, *p-*value = 0.003), sepsis (OR 2.98, 95% CI: 1.99–4.46, *p-*value< 0.001), septic shock (OR 4.51, 95% CI: 2.83–7.23, *p-*value< 0.001), placement of foley catheter (OR 4.24, 95% CI: 2.75–6.61, *p-*value< 0.001), and placement of endotracheal tube (OR 6.98, 95% CI: 4.54–10.73, *p-*value< 0.001) (Table 2).

**TABLE 2:** | Association between factors and mortality using univariate analysis.

Factors	Total (*n* = 504)		
	Odds ratio	95% CI	*p-*value
Male	1.25	0.84–1.85	0.29
Aged ≥ 65 years	1.12	0.76–1.67	0.568
Hospitalization > 14 days	1.88	1.27–2.78	0.001*
Admission in ICU	1.70	1.10–2.61	0.014*
Bedridden	1.34	0.77–2.32	0.279
Immunocompromised host	1.76	1.19–2.60	0.004*
Hypertension	1.14	0.77–1.67	0.512
Type 2 Diabetes mellitus	1.03	0.66–1.58	0.916
Dyslipidemia	0.79	0.47–1.30	0.343
Heart failure	3.48	1.23–10.62	0.012*
Chronic kidney disease	1.14	0.74–1.74	0.600
Atrial fibrillation	3.31	1.48–7.67	0.002*
Chronic obstructive pulmonary disease	2.78	1.44–5.42	0.002*
Gout	0.56	0.24–1.20	0.132
Liver cirrhosis	1.52	0.47–4.68	0.429
Coronary artery disease	0.86	0.34–2.03	0.841
Neurological disease	1.97	0.58–6.69	0.254
Cerebrovascular disease	0.89	0.42–1.79	0.868
Mental disorder	0.64	0.06–3.62	0.722
Bacteremia	0.32	0.21–0.48	<0.001*
Urinary tract	2.16	1.33–3.49	0.001*
Pneumonia	7.11	3.63–14.56	<0.001*
Wound	0.85	0.40–1.75	0.735
Hospitalization within 3 months	1.34	0.91–1.97	0.132
Surgery within 3 months	0.65	0.26–1.47	0.356
Use of antibiotics within 3 months	1.77	1.19–2.61	0.003*
Sepsis	2.98	1.99–4.46	<0.001*
Septic shock	4.51	2.83–7.23	<0.001*
Foley catheter	4.24	2.75–6.61	<0.001*
Endotracheal tube	6.98	4.54–10.73	<0.001*

*Factor was significant. Abbreviations: Cl, confidence interval; ICU, intensive care unit.

### Model development

**FIGURE 2:**
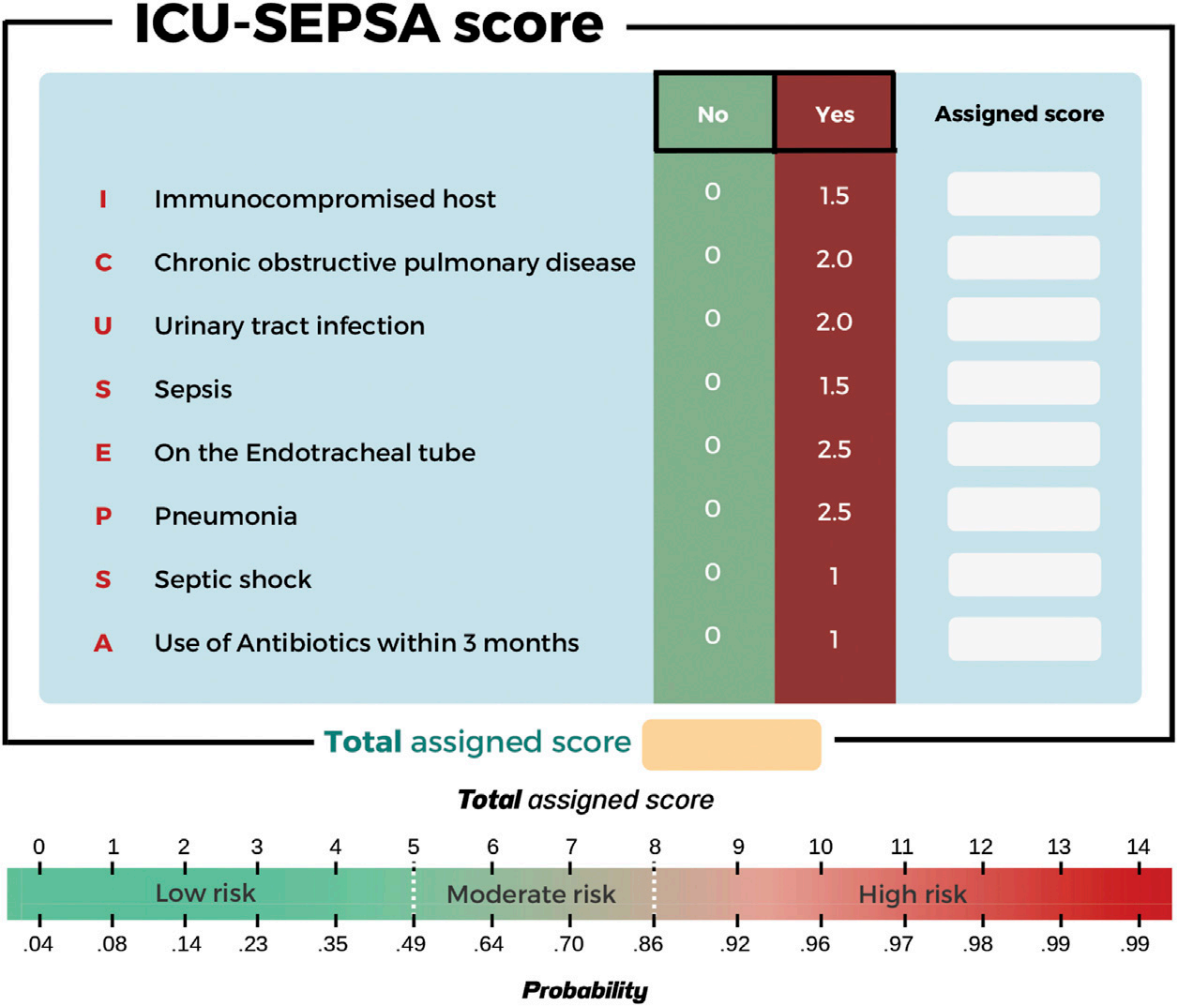
Visualize used for predicting mortality in patients with MDR infection.

In the multivariable analysis, some variables were independent predictors of mortality. These were chronic obstructive pulmonary disease (OR 3.59, 95% CI:1.61–7.99, *p*-value = 0.002), pneumonia (4.86, 95% CI: 2.29–10.27, *p*-value<0.001), urinary tract infection (OR 3.24, 95% CI: 1.85–5.66, *p-*value<0.001), use of antibiotics within the past 3 months (OR 1.89, 95% CI: 1.18–3.01, *p-*value = 0.008), immunocompromised host (OR 2.24, 95% CI: 1.39–3.62, *p-*value = 0.001), sepsis (OR 2.24, 95% CI: 1.31–3.82, *p-*value = 0.003), septic shock (OR 2.02, 95% CI: 1.09–3.74, *p-*value = 0.026), and placement of endotracheal tube (OR 5.30, 95% CI: 3.27–8.59, *p-*value<0.001) (Table 3 and Figure 2). Since the VIF values of each variable were less than 5 (averaged value was 1.17), multi-collinearity was not a major identified in our study. We have analyzed the AIC and BIC scores to help identify variables that would be incorporated in the predictive model. The model with the lowest AIC and BIC values was chosen as the best model for predicting mortality in patients with MDR infection (Supplementary Table S2).

**TABLE 3:** | Significant factors of mortality in patients with MDROs using multivariable analysis (ICU-SEPSA score).

Predictors	Coefficient	*p-*value	Transformed coefficients	Assigned score
Immunocompromised host				
No	—	—	—	0
Yes	0.760152	0.001	1.27	1.5
Chronic obstructive pulmonary disease				
No	—	—	—	0
Yes	1.204286	0.002	2.01	2
Urinary tract infection				
No	—	—	—	0
Yes	1.108349	<0.001	1.85	2
Sepsis				
No	—	—	—	0
Yes	0.758534	0.003	1.27	1.5
On the Endotracheal tube				
No	—	—	—	0
Yes	1.571675	<0.001	2.63	2.5
Pneumonia				
No	—	—	—	0
Yes	1.489380	<0.001	2.49	2.5
Septic shock				
No	—	—	—	0
Yes	0.661689	0.026	1.11	1
Use of Antibiotics within 3 months				
No	—	—	—	0
Yes	0.598458	0.008	1	1

The coefficient of model intercept is -3.010593 (*p*-value <0.001).

**FIGURE 3:**
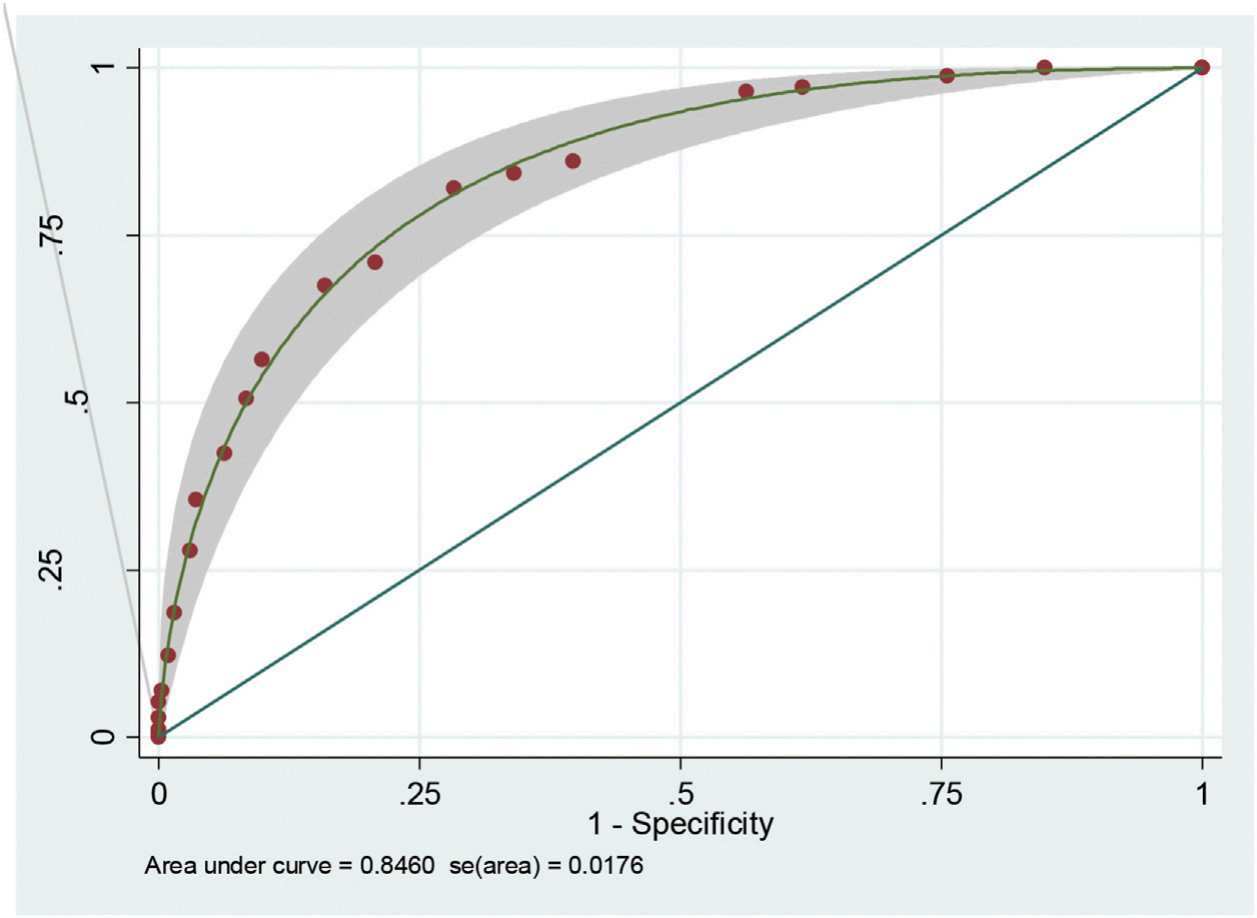
Receiver operating characteristic (ROC) curve of risk scoring system.

**FIGURE 4:**
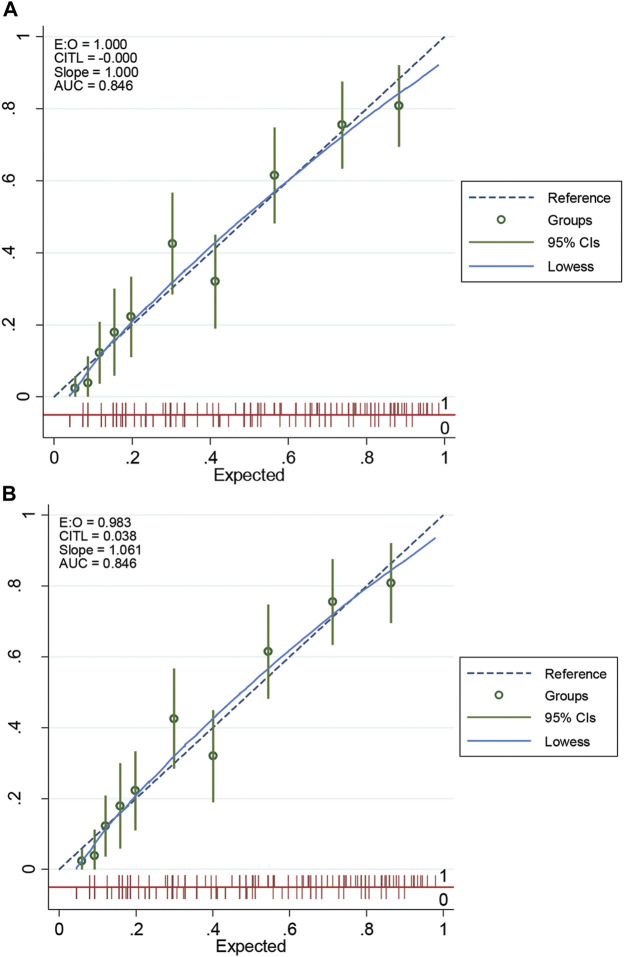
The calibration curve of original performance expressed in **(A)**; Internally validated performance expressed in **(B)**. EO: expected-observed ratio; CITL: calibration-in-the-large; AUC: area under the receiver operating characteristic curve; CI: confidence interval.

**FIGURE 5:**
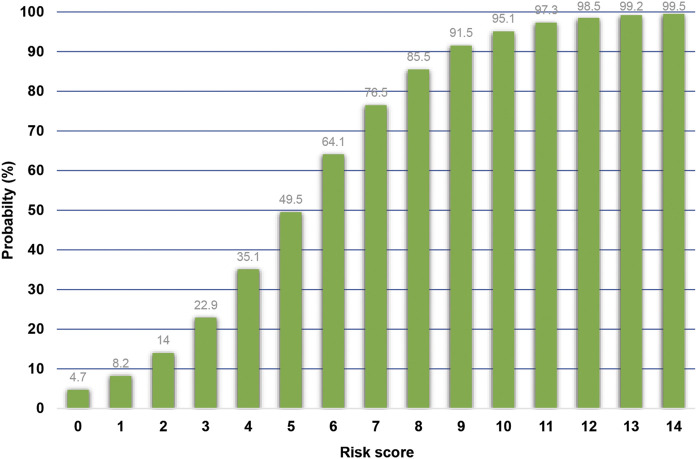
Probability of mortality in patients with MDROs stratified by the score.

This scoring system was created by setting the cut-off point according to the differentiation plot and performance of diagnostic parameters to identify patients at risk of increased mortality. Cut-off scores of 5 and 8 were chosen to classify patient into 3 groups (Table 4). Patients with a score ≥ 8 were placed in the high-risk group. Patients in this group were correctly predicted for the mortality outcome 86.49% (32/37) of the time. Patients with a score ≤ 5 were placed in the low-risk group and correctly predicted for the mortality outcome 79.95% (299/374) of the time. Correct prediction of survival or death occurred 80.54% [(299 + 32)/(374 + 37)] of the time. The incorrect prediction rate was 19.46% [(75 + 5)/(374 + 37)]. Based on this scoring system and one cut-off point, the original performance model showed good discrimination (AUC = 84.60%, 95%Cl: 81%–88%; Hosmer-Lemeshow χ2 = 19.27; *p-*value = 0.50) and calibration (calibration slope, 1.000; Brier score, 0.149; product moment correlation between observed and predicted probability, 1.000). Internally validated performance that gave an AUC similar original performance model and calibration (calibration slope, 1.061; Brier score, 0.149; product moment correlation between observed and predicted probability, 0.983) (Figures 3, 4A,B). (Marcin and Romano, 2007) The probability of mortality is presented in Figure 5.

**TABLE 4:** Distribution of the risk of mortality in patients with MDROs and diagnostic performance.

Parameters	Low risk (score ≤ 5)	Moderate risk (score 5.5–7.5)	High risk (score ≥ 8)	Total (*n* = 504)
Total	374	93	37	504
Survival group	299	28	5	332
Death group	75	65	32	172
Diagnostic performance (95% Confidence Interval)				
Sensitivity (%)	90.06 (86.32–93.06)		18.60 (13.09–25.24)	
Specificity (%)	56.40 (48.64–63.93)		98.49 (96.52–99.51)	
Positive predictive value (%)	79.95 (77.02–82.59)		86.49 (71.75–94.16)	
Negative predictive value (%)	74.62 (67.45–80.65)		70.02 (68.47–71.52)	
Accuracy	78.57 (74.73–82.08)		71.23 (67.06–75.15)	
Likelihood ratio (+)	2.07 (1.74–2.46)		12.35 (4.90–31.13)	
Likelihood ratio (-)	0.18 (0.12–0.25)		0.83 (0.77–0.89)	
Interpretation	Survive (74.62% certainty)		Death (86.49% certainty)	

## Discussion

Due to lack of predictive scoring for mortality in patients with multidrug resistant infection, we aimed to develop a risk scoring model to predict mortality in this population. Our study revealed that a clinical scoring system could be used to screen and predict the mortality in patients with MDROs. Our newly formulated tool, named “ICU-SEPSA score”, was able to classify patients at risk of mortality.

During our study, we collected data of 504 patients from October 2018 to September 2020. Using newly identified eight factors, we were able to categorize patients into three groups in relation to mortality risk: low, moderate, and high. These factors were 1) Immunocompromised host, 2) chronic obstructive pulmonary disease, 3) urinary tract infection, 4) sepsis, 5) placement of endotracheal tube, 6) pneumonia, 7) septic shock, and 8) use of antibiotics within the past 3 months. We observed similar findings compared to previous studies. (Miravitlles et al., 1999; Johnson et al., 2011; Lee et al., 2016; Moreau et al., 2018; Capsoni et al., 2019; Cillóniz et al., 2019; Du et al., 2019) While the exact relationship between immunocompromised hosts and mortality is unknown, the study by Moreau et al. (2018) found that immunocompromised hosts were linked to recent use of antibiotics. This is a well-known risk factor for developing MDROs and increased risk of mortality. In our study, we found that sepsis and septic shock were associated with higher mortality rates due to multiple organ dysfunction. (Capsoni et al., 2019) Use of any medical devices that posed an increased risk of bacteria growth either through lower bacteria clearance or epithelial damage that allowed formation of biofilm, can result in higher risk of mortality. In our study, we found that endotracheal tube was associated with mortality in MDROs infections. (Nseir et al., 2006) In addition, a previous systematic review and meta-analysis showed that intubation was considered as an invasive techniques associated with adverse outcomes. (Du et al., 2019) It has been established that some comorbidities are associated with mortality, particularly chronic obstructive pulmonary disease, but the mechanism is unclear. A previous study suggested that patients with acute exacerbation of chronic obstructive pulmonary disease required intubation or ventilators more often. (Nseir et al., 2006) The sputum culture of patients with severe pulmonary function impairment, indicated by forced expiratory volume in one second (FEV-1) <50%, has shown to contain more potentially pathogenic microorganisms, such as *Haemophilus influenzae* or *P. aeruginosa*. (Miravitlles et al., 1999) In our study, we found that certain types of infections (urinary tract infection and pneumonia) led to increased risks of mortality. This may be the result of receiving insufficient empiric treatment leading to the development of MDROs infections. (Lee et al., 2016; Cillóniz et al., 2019) The last factor correlated to mortality in patients with MDR pathogens was the use of antibiotics within the past 3 months. We hypothesized that prior antibiotic use led to higher likelihood of antimicrobial resistance. (Johnson et al., 2011)

Our study has a number of strengths. To the extent of our knowledge, this was the first time that risk scoring tool to predict mortality secondary to any MDROs infections was identified. Our data analysis was not restricted to a particular type of infections. We evaluated various sources of cultures, including blood, urine, sputum, catheter, ascitic fluid and pus. Our tool was user friendly. It was simple and did not require any specific or expensive laboratory investigation. The newly established tool, named “ICU-SEPSA score” consisted of eight predictors, is easy to remember for clinicians. We foresee a great impact of this tool to help assess mortality risks in MDRO-infected patients. Lastly, the power of this model was sufficient to detect a difference between the two groups.

There are several limitations to this study. First, the data was collected retrospectively from medical records and electronic database. There was a potential absence of information that could impact data analysis and outcome such as type of organism, smoking status, history of alcohol use, and pertinent past medical history. In addition, during medical record review, we were unable to assess the severity of some variables such as urinary tract infection and pneumonia. Third, this study was designed as a single-center study, therefore, the ability to generalize our results may be limited. Lastly, it is worth noting that our model has not been conducted in the external validation cohort.

Our newly formulated tool may be incorporated into clinical practice to help healthcare providers estimate the risk of mortality in this population. A high score corresponds to a greater risk of mortality in patients with MDROs infections that should assist clinicians to promptly identify the appropriate therapy. To implement our findings in clinical setting, we suggested that patients who classified into the high-risk group (score ≥ 8) should be considered for admission in the ICU for close monitoring. These patients should receive combination antibiotic therapy for synergistic effect and reduced risk of modified risk factors. Frequent vital sign monitoring is warranted for moderate-risk group (score 5.5–7.5) as well as the high-risk group. At Phrae Hospital, vital sign monitoring as frequently as every 2 h had been suggested. In addition, every effort to minimize prolonged use of any catheters should be considered. Patients in the low-risk group (score ≤ 5) should still have vital signs monitored regularly according to the standard of care at that clinical setting. Regardless of which group patients are categorized into, MDROs infections should be immediately assessed. Aggressive therapy with close monitoring is key to lower risks of mortality and optimize overall outcome.

## Conclusion

A simplified predictive scoring tool wad developed to predict mortality in patients with MDROs infections. The score was composed of eight factors including 1) immunocompromised host, 2) chronic obstructive pulmonary disease, 3) urinary tract infection, 4) sepsis, 5) placement of endotracheal tube, 6) pneumonia, 7) septic shock, and 8) use of antibiotics within the past 3 months. Due to a single-study design of this study, external validation of the results before applying in other clinical practice settings is warranted.

## Data Availability

The original contributions presented in the study are included in the article/Supplementary Material, further inquiries can be directed to the corresponding authors.

## Abbreviations

AuROC, The area under the receiver operating characteristic curve; CITL, calibration-in-the-large; EPV, events per predictor variable; FEV1, forced expiratory volume in one second; ICD-10, the International Classification of Diseases, 10th Revision, Clinical Modification; ICU, intensive care unit; LR+, positive likelihood ratio; LR-, negative likelihood ratio; MDR, multidrug-resistant; MDROs, multidrug-resistant organisms; NPV, negative predictive value; ORs, odds ratios; PPV, positive predictive value; ROC, receiver operating characteristic; TRIPOD, the Transparent Reporting of a multivariable prediction model for Individual Prognosis Or Diagnosis; 95%CI, 95% confidence intervals.
